# Mechanism of Breast Cancer Preventive Action of Pomegranate: Disruption of Estrogen Receptor and Wnt/β-Catenin Signaling Pathways

**DOI:** 10.3390/molecules201219853

**Published:** 2015-12-12

**Authors:** Animesh Mandal, Anupam Bishayee

**Affiliations:** 1 Department of Pharmaceutical Sciences, College of Pharmacy, Northeast Ohio Medical University, Rootstown, OH 44272, USA; animandal@rediffmail.com; 2 Department of Pharmaceutical Sciences, College of Pharmacy, Larkin Health Sciences Institute, 18301 N. Miami Avenue, Miami, FL 33169, USA

**Keywords:** mammary tumorigenesis, DMBA, pomegranate (*Punica granatum* L.), estrogen receptors, β-catenin, cyclin D1

## Abstract

A pomegranate emulsion (PE), containing various bioactive phytochemicals, has recently been found to exert substantial chemopreventive effect against 7,12-dimethylbenz(*a*)anthracene (DMBA)-induced mammary tumorigenesis in rats via antiproliferative and proapoptotic actions. Nevertheless, the underlying mechanisms of action are not completely understood. The present study was designed to investigate the effects of PE treatment on intratumor expression of estrogen receptor (ER)-α, ER-β,β-catenin and cyclin D1 during DMBA rat mammary carcinogenesis. Mammary tumor sections were harvested from a chemopreventive study in which PE (0.2, 1.0 and 5.0 g/kg) exhibited inhibition of mammary tumorigenesis in a dose-response manner. The expressions of ER-α, ER-β, β-catenin and cyclin D1 were analyzed by immunohistochemical techniques. PE downregulated the expression of intratumor ER-α and ER-β and lowered ER-α:ER-β ratio. PE also decreased the expression, cytoplasmic accumulation, and nuclear translocation of β-catenin, an essential transcriptional cofactor for Wnt signaling. Moreover, PE suppressed the expression of cell growth regulatory protein cyclin D1, which is a downstream target for both ER and Wnt signaling. Our current results in conjunction with our previous findings indicate that concurrent disruption of ER and Wnt/β-catenin signaling pathways possibly contributes to antiproliferative and proapoptotic effects involved in PE-mediated chemoprevention of DMBA-inflicted rat mammary tumorigenesis.

## 1. Introduction

Breast cancer is the most common cancer among women and the second leading cause of cancer-associated deaths in humans worldwide. According to GLOBOCAN 2012 [1], 522,000 women died of breast cancer in both developed and developing countries in 2012. In 2015, approximately 232,000 new breast cancer cases and about 40,000 deaths are estimated to occur in women in the United States [2]. Interestingly, breast cancer also occurs in men with very less frequency compared to women [3]. Genetic risk factors, including mutations on breast cancer susceptibility gene 1 (*BRCA1*) and *BRCA2*, are responsible for about 5%–10% of all breast cancer incidences [4,5]. There are several acquired risk factors for breast cancer which include early onset of menstruation, not having children, delayed birth of a first child, short duration of breast feeding, late menopause, use of hormone replacement therapy, aging, obesity, diabetes, alcohol consumption and circadian disruption [6,7,8,9,10,11,12,13].

Current treatment options for early-stage breast cancer include surgical resection, radiotherapy and adjuvant chemotherapy and/or hormone therapy. Although there are a large numbers of chemotherapeutic drugs, development of resistance and severe adverse side effects represent two serious challenges in the management of breast cancer using these drugs. These facts underscore the importance of developing novel drugs which are more effective and less toxic. Another prudent approach could be chemoprevention which represents prevention of breast cancer occurrence through dietary means and/or use of pharmacological and natural agents [6,7,14,15]. Various epidemiological studies have shown that consumption of high levels of fruits, vegetables and beverages reduce the risk of breast cancer development and recurrence as well as increase the survival rate of patients with this cancer [16,17,18,19,20,21]. Numerous bioactive phytochemicals present in dietary and non-dietary agents have been found to kill breast tumor cells *in vitro* and suppress the development of mammary tumors or retard the growth of existing tumors *in vivo* through modulation of proliferation, differentiation, apoptosis, oxidative stress, inflammation, neovascularization, and several important cell signaling pathways [22,23,24,25,26,27,28,29]. Moreover, several clinical intervention trials investigated the potential efficacy of various dietary supplements and natural products in breast cancer prevention and treatment [30,31].

Pomegranate (*Punica granatum*, L.), an ancient, mystical and highly distinctive fruit, is widely consumed in various parts of the world. Pomegranate fruit has been gaining widespread popularity as a functional food and nutraceutical source due to published reports on potential health benefits, including prevention and/or treatment of oncologic diseases, cardiovascular and neurological disorders, inflammation, ulcer, arthritis, microbial infection, obesity, diabetes, acquired immune deficiency syndrome, and male infertility [32,33,34,35]. Pomegranate fruit represents a rich reservoir of phytochemicals, including polyphenols, hydrolysable tannins, (punicalagin, ellagic acid, gallic acid and gallagic acid), fatty acid (punicic acid) and anthocyanins (delphinidin, cyaniding and pelargonidin). Various preclinical and clinical studies demonstrate chemopreventive and therapeutic effects of pomegranate-derived substances, such as juice, extracts and phytochemicals, against prostate, colon, lung, and skin cancer [36,37]. Based on numerous *in vitro* studies, several pomegranate products and phytoconstituents exhibited cytotoxic, antiproliferative, proapoptotic, antiangiogenic, anti-invasive, and antimetastatic effects against estrogen receptor-positive and -negative breast carcinoma cells [38,39,40,41,42,43,44,45,46,47,48]. Pomegranate seed oil and fermented juice concentrate suppressed 7,12-dimethyl benz(*a*)anthracene (DMBA)-induced precancerous mammary gland lesions *ex vivo* [49] and pomegranate extract inhibited the growth of xenografted BT-474 tumors *in vivo* [45].

Recently, we have reported for the first time that a pomegranate formulation (emulsion) containing most bioactive phytochemicals present in the whole fruit affords a remarkable chemopreventive effect against DMBA-induced mammary tumorigenesis in rats [50]. In this study, pomegranate emulsion (PE) reduced the incidence, total burden and average weight of mammary tumors in DMBA-initiated rats with a concurrent inhibition of cell proliferation, induction of apoptosis, upregulation of proapoptotic protein Bax, and downregulation of antiapoptotic protein Bcl-2 in mammary tumors [50]. Since estrogen receptors (ERs) are involved in mammary cell proliferation [51,52] and DMBA-inflicted rat mammary tumors express ERs [53], we hypothesize that PE-mediated inhibition of mammary tumor cell proliferation could be attained via interference with the expressions of ERs. Moreover, since upregulation of Wnt/β-catenin signaling, which plays a pivotal function in regulation of cell proliferation and apoptosis, has been observed in DMBA-induced mammary tumors in rats [54], it is conceivable that PE could impart antiproliferative and proapoptotic effects through disruption Wnt/β-catenin signaling and thereby blocking expression of downstream genes responsible for promotion of cell proliferation and suppression of apoptotic cell death during rat mammary carcinogenesis. Accordingly, the present study was conducted to extend our previous work [50] to investigate the effects of PE treatment on ER and Wnt/β-catenin signaling as well as expression of cyclin D1, a downstream target for both ER and Wnt signaling, during DMBA-initiated rat mammary tumorigenesis.

## 2. Results

### 2.1. PE Suppresses Elevated ER-α and ER-β Expressions during DMBA-Induced Mammary Tumorigenesis

**Figure 1:**
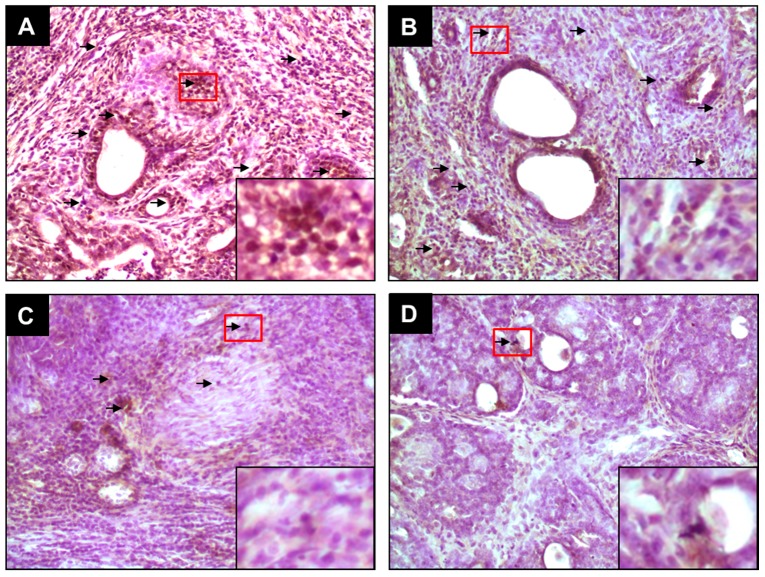
Effect of PE on expression of ER-α during DMBA-induced rat mammary gland tumorigenesis. The rats were treated with various oral doses of PE (three times a week) 2 weeks prior to and 16 weeks after DMBA administration. All animals were sacrificed 16 weeks post-DMBA treatment. The mammary tumors were subjected to immunohistochemical analysis using anti-ER-α antibody. Arrows indicate immunohistochemical staining of ER-α (magnification: ×200). The nuclear expression of ER-α in the designated area marked by red box is shown as an inset (magnification: ×1000) for each treatment group. Various treatment groups are: (**A**) DMBA control; (**B**) PE at 0.2 g/kg body weight plus DMBA; (**C**) PE at 1.0 g/kg body weight plus DMBA; and (**D**) PE at 5.0 g/kg body weight plus DMBA.

**Figure 2:**
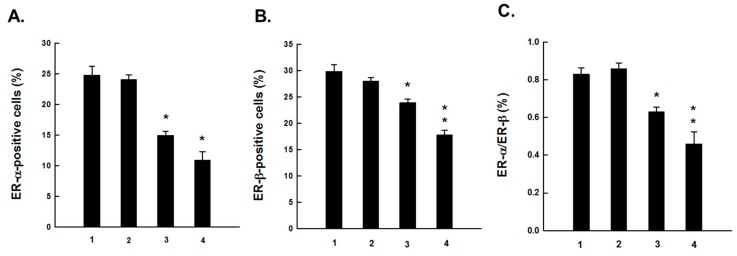
Quantitative analysis of (**A**) ER-α-immunopositive cells; (**B**) ER-β-immunopositive cells and (**C**) ER-α/ER-β ratio during DMBA mammary carcinogenesis in rats in the presence or absence of PE treatment (0.2, 1.0 or 5.0 g/kg). Results are based on 1000 cells per animal and 4 animals per group. Various experimental groups are: (1) DMBA control; (2) PE (0.2 g/kg) + DMBA; (3) PE (1.0 g/kg) + DMBA; and (4) PE (5.0 g/kg) + DMBA. Each bar represents the mean ± SEM (*n* = 4). (**A**) * *p* < 0.001; (**B**) * *p* < 0.01 and ** *p* < 0.001 and (**C**) * *p* < 0.05 and ** *p* < 0.001 as compared to DMBA control.

**Figure 3:**
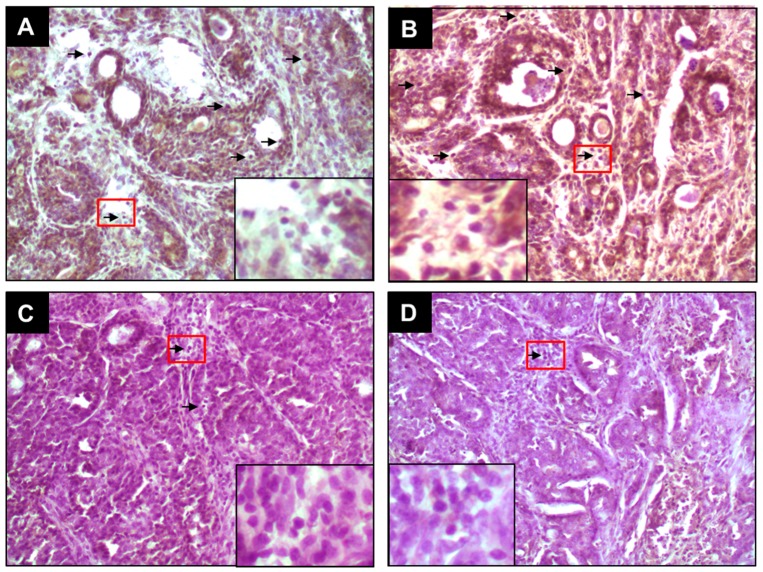
Effect of PE on expression of ER-β during DMBA-induced rat mammary gland tumorigenesis. The rats were treated with various oral doses of PE (three times a week) 2 weeks prior to and 16 weeks after DMBA administration. All animals were sacrificed 16 weeks post-DMBA treatment. The mammary tumors were subjected to immunohistochemical analysis using anti-ER-β antibody. Arrows indicate immunohistochemical staining of ER-β (magnification: ×200). The nuclear expression of ER-β in the designated area marked by red box is shown as an inset (magnification: ×1000) for each treatment group. Various treatment groups are: (**A**) DMBA control; (**B**) PE at 0.2 g/kg body weight plus DMBA; (**C**) PE at 1.0 g/kg body weight plus DMBA; and (**D**) PE at 5.0 g/kg body weight plus DMBA.

Since ER status is an important classifier of breast cancer, intratumor expressions of ER-α and ER-β in DMBA-initiated rats in the presence or absence of PE treatment were investigated using immunohistochemical techniques. The protein expression of ER-α and ER-β was detected chiefly in the nuclei of epithelial cells. The frequency and intensity of ER-α-immunopositive cells were very high in tumor sections harvested from DMBA control animals (Figure 1A). PE, at 0.2 g/kg, did not alter the expression pattern of ER-α in tumors from DMBA-initiated rats (Figure 1B). On the contrary, a dose-responsive decrease in the expression of ER-α was noticed in tumor sections harvested from animals treated with 1.0 g/kg (Figure 1C) or 5.0 g/kg (Figure 1D) of PE compared to DMBA control. The quantitative analysis reveals immunopositivity for nearly 25% of mammary tumors cells in DMBA control rats (Figure 2A). A significant (*p* < 0.001) reduction in the percentage of ER-α-positive tumor cells in rats treated with 1.0 g/kg or 5.0 g/kg of PE was noticed compared to DMBA control (Figure 2A). Like ER-α, an ample expression of ER-β was observed in tumor samples of rats exposed to DMBA alone (Figure 3A). Although the expression of ER-β was not altered by PE at 0.2 g/kg (Figure 3B), a dose of 1.0 (Figure 3C) or 5.0 g/kg (Figure 3D) displayed considerable attenuation of ER-β immunopositivity. The quantitative analyses of immunopositive cells indicated a significant (*p* < 0.01 and *p* < 0.001) reduction in ER-β-positive cells (Figure 2B) in tumor samples from rats that received 1.0 and 5.0 g/kg PE compared to DMBA control, respectively. These doses of PE attenuated the ratio of ER-α to ER-β in a statistically significant (*p* < 0.05 or 0.001) manner.

### 2.2. PE Interferes with Activated β-Catenin Signaling during Mammary Tumorigenesis Induced by DMBA

**Figure 4:**
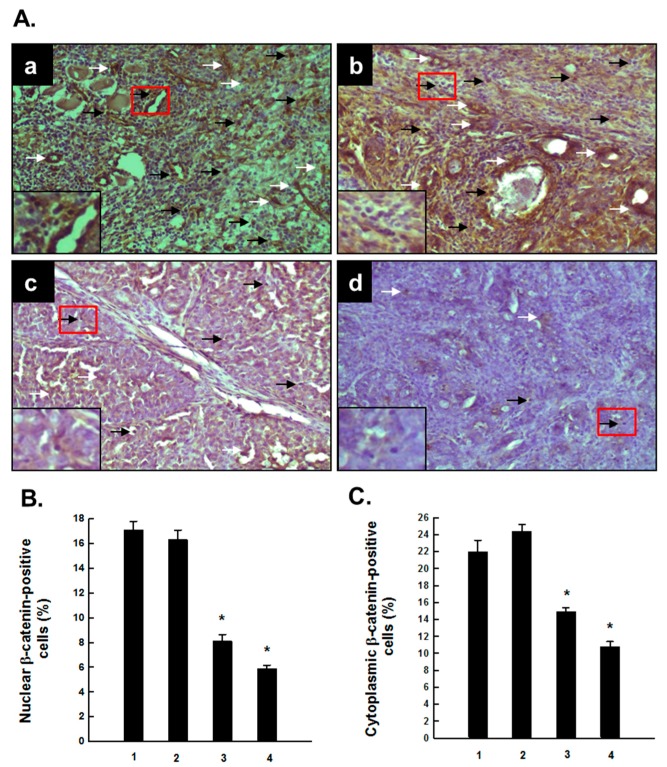
Effect of PE on expression β-catenin during DMBA-induced mammary carcinogenesis in rats. (**A**) Immunohistochemical detection of β-catenin in several experimental rat groups. The rats were treated with various oral doses of PE (three times a week) 2 weeks prior to and 16 weeks after DMBA administration. All animals were sacrificed 16 weeks post-DMBA treatment. The mammary tumors were subjected to immunohistochemical analysis using anti-β-catenin antibody. Representative immunohistochemical localization of β-catenin in nucleus (black arrows) and cytosol (white arrows) is depicted (magnification: ×200). The nuclear expression of β-catenin in the designated area marked by red box is shown as an inset (magnification: ×1000) for each treatment group. Various treatment groups are: (a) DMBA control; (b) PE at 0.2 g/kg body weight plus DMBA; (c) PE at 1.0 g/kg body weight plus DMBA; and (d) PE at 5.0 g/kg body weight plus DMBA; (**B**) Quantitative analysis of nuclear and (**C**) cytoplasmic β-catenin-immunopositive cells in rat mammary tumors induced by DMBA in the presence or absence of PE treatment (0.2, 1.0 or 5.0 g/kg). Results are based on 1000 cells per animal and 4 animals per group. Each bar represents the mean ± SEM (*n* = 4). * *p* < 0.001 as compared to DMBA control. Various experimental groups are: (1) DMBA control; (2) PE (0.2 g/kg) + DMBA; (3) PE (1.0 g/kg) + DMBA; and (4) PE (5.0 g/kg) + DMBA.

As depicted in Figure 4A, the immunohistochemical profile indicates alteration in nuclear and cytosolic expressions of β-catenin in tumor sections harvested from several animal groups exposed to DMBA. Substantial expression of both nuclear and cytosolic β-catenin-positive cells was observed in rats subjected to DMBA mammary carcinogenesis (Figure 4A-a). The rats which had oral PE at 0.2 g/kg in addition to DMBA showed minimal changes in the expression of nuclear as well as cytosolic expression of β-catenin compared to DMBA control (Figure 4A-b). A considerable decrease in the expression of β-catenin in both nucleus and cytoplasm was achieved by PE treatment at a dose of either 1.0 (Figure 4A-c) or 5.0 g/kg (Figure 4A-d). The accompanying quantitative analysis (Figure 4B,C) confirms our immunohistochemical results, depicting a significant (*p* < 0.001) decrease in nuclear and cytosolic β-catenin expression in rats treated with PE at 1.0 or 5.0 g/kg plus DMBA compared to DMBA alone.

### 2.3. PE Downregulates Cyclin D1 Expression during DMBA-Induced Mammary Carcinogenesis

**Figure 5:**
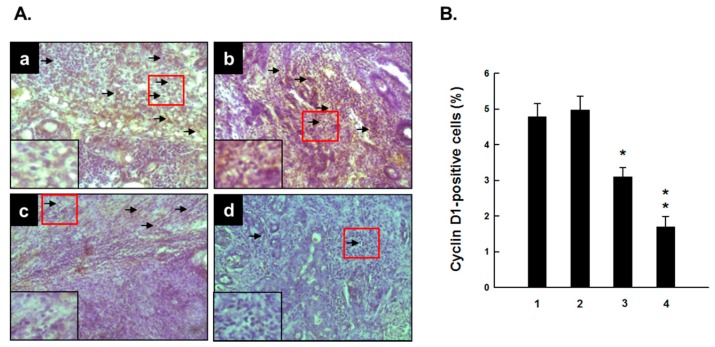
Effect of PE on expression of cyclin D1 during DMBA-inflicted mammary gland tumorigenesis in rats. (**A**) Immunohistochemical detection of cyclin D1 in several experimental rat groups. The rats were treated with various oral doses of PE (three times a week) 2 weeks prior to and 16 weeks after DMBA administration. All animals were sacrificed 16 weeks post-DMBA treatment. The mammary tumors were subjected to immunohistochemical analysis using anti-cyclin D1 antibody. Arrows indicate immunohistochemical localization of cyclin D1 in nucleus (magnification: ×200). The nuclear expression of cyclin D1 in the designated area marked by red box is shown as an inset (magnification: ×1000) for each treatment group. Various treatment groups are: (a) DMBA control; (b) PE at 0.2 g/kg body weight plus DMBA; (c) PE at 1.0 g/kg body weight plus DMBA; and (d) PE at 5.0 g/kg body weight plus DMBA; (**B**) Quantitative analysis of cyclin D1-immunopositive cells in rat mammary tumors induced by DMBA in the presence or absence of PE treatment (0.2, 1.0 or 5.0 g/kg). Results are based on 1000 cells per animal and 4 animals per group. Each bar represents the mean ± SEM (*n* = 4). * *p* < 0.05 and ** *p* < 0.001 as compared to DMBA control. Various experimental groups are: (1) DMBA control; (2) PE (0.2 g/kg) + DMBA; (3) PE (1.0 g/kg) + DMBA; and (4) PE (5.0 g/kg) + DMBA.

Figure 5 illustrates immunohistochemical results showing expression of cell cycle specific gene cyclin D1 in mammary tumor samples from various experimental animal groups. Cyclin D1 was found to be expressed predominantly in the nuclei of tumor cells in DMBA control animals (Figure 5A-a). A marginal alteration in the expression of cyclin D1 was observed in the group that received 0.2 g/kg PE plus DMBA compared to DMBA control (Figure 5A-b). On the other hand, tumor sections from rats that had PE at 1.0 (Figure 5A-c) and 5.0 g/kg (Figure 5A-d) exhibited moderate and substantial reduction in the expression of cyclin D1, respectively. The quantitative analysis indicates a marginal increase in the percentage of cyclin D1-immunopositive cells in the group treated with 0.2 g/kg PE compared to DMBA control (Figure 5B). However, a significant (*p* < 0.05 or 0.001) decrement in the percentage of cyclin D1-positive cells was achieved in DMBA-initiated rats treated with PE at 1.0 or 5.0 g/kg in comparison with DMBA control, respectively (Figure 5B).

## 3. Discussion

Elevated lifetime exposure to endogenous or exogenous estrogen has been accepted as the single most important risk factor in the development of breast cancer [55]. Estrogen activation of ERs triggers specific signaling pathways responsible for mammary cell proliferation and differentiation and plays a pivotal role in the development of healthy mammary glands. On the other hand, altered ER signaling is associated with abnormal cell proliferation as well as initiation and progression of breast cancer [51,52]. Emerging evidence suggest that 70% of breast cancers express ERs [56]. It is well known that estrogen binds to ER-α and ER-β and activates transcription of estrogen-responsive genes, resulting in accelerated tumor cell proliferation [57,58]. Accordingly, the modulation of ER-α and ER-β may be a central mechanism to suppress mammary gland carcinogenesis [59,60]. Use of antiestrogenic drugs capable of competing with estrogen for binding to ERs is known to be effective in impeding estrogen-dependent mammary tumor growth as well as preventing the occurrence of breast tumor [14,61,62].

In our study, we have evaluated the expression of ER-α and ER-β in DMBA-induced mammary tumors in rats subjected to PE treatment. Our results indicate considerable expression of ER-α and ER-β in mammary tumors harvested from DMBA control animals. The oral administration of PE to rats before and after DMBA treatment decreased both ER-α and ER-β protein expression in mammary tumors. PE may reduce ER gene transcription, translation or induce epigenetic modifications. However, additional studies are warranted to confirm the exact mechanism of reduced levels of ER-α and ER-β in DMBA-induced tumors in PE treatment groups. Another salient feature of our study is PE-mediated decrement in the ratio of ER-α to ER-β. An earlier report documents an increase in the ERα:ER-β in human breast tumorigenesis [59]. Moreover, an elevated ERα:ER-β correlates with higher level of cell proliferation in preinvasive human mammary tumors [63]. Phytoconstituents present in PE may compromise the responsiveness to endogenous estrogen by diminishing the expression of ERs, resulting reduced availability of nuclear receptor sites for estrogen binding during experimental mammary carcinogenesis. The overall effect could be a significant reduction in intratumor proliferation by a reduced expression of proliferating cell nuclear antigen as we reported recently [50]. Furthermore, the prior exposure of the mammary glands to PE may compromise the ability of ER-positive cells to respond to DMBA challenge. Collectively, all these attributes could be responsible for subsequent development of fewer proliferating mammary tumors as we have observed in our previous study [50]. Interestingly, our results are in line with a previous report showing that a methanolic extract of pericarp (peel) of pomegranate inhibited the binding of estradiol to ER, downregulated ER-α gene, and suppressed the growth and proliferation of ER-positive MCF-7 breast cancer cells [47].

It is likely that estrogens impart their oncogenic effects through regulation of ER-dependent as well as ER-independent pathways. Therefore, the net mammary tumor inhibitory effect of PE in DMBA-initiated, estrogen-dependent mammary carcinogenesis model could be attributed to PE-mediated disruption of ER signaling as well as non-ER mediated mechanisms. As a matter of fact, several pomegranate-derived products and phytochemicals inhibited the growth of ER-negative MBA-MD-231 breast cancer cells [38,45].

The canonical Wnt or Wnt/β-catenin signaling pathway plays an important role in mammary gland development as well as tumorigenesis due to its involvement in signal transduction, cellular adhesion, and regulation of cell-context-specific gene expression [64,65]. In an absence of a Wnt signal, β-catenin is linked to a multiprotein complex, consists of Axin, adenomatous polyposis coli, casein kinase-1α, and glycogen synthase kinase, which facilitates its phosphorylation, ubiquitination and degradation by proteasome [66]. In the presence of a Wnt signal, the destruction complex is inactivated, which leads to stabilization and accumulation of β-catenin in the cytoplasm. Consequently, β-catenin translocates to the nucleus and binds to T-cell factor/lymphoid-enhancer factor, resulting in activation of transcription of various target genes, such as c-myc, cyclin D1, matrix metalloproteinase 7, and vascular endothelial growth factor, which are implicated in mammary gland carcinogenesis [67]. A significant accumulation of β-catenin in the nucleus and/or cytoplasm has been observed in human breast carcinoma samples and is believed to be associated with poor prognosis [68,69]. Interestingly, upregulation of nuclear and/or cytoplasmic β-catenin in human breast cancer samples has been correlated with the expression of its target gene - cyclin D1 [70]. Additionally, an elevated accumulation of cytosolic and nuclear β-catenin has been observed in ductal carcinoma *in situ* and basal-like *in situ* breast tumors, indicating that activation of Wnt/β-catenin pathway may be an early event in human breast cancer [68,71]. Consistent with clinical situations, an increase in total and nuclear β-catenin protein has been found in DMBA-induced mammary tumors in mice, showing stimulation of this oncogenic signaling pathway [72]. Furthermore, a sequential elevation of β-catenin level in mammary tissues have been found in rats subjected to DMBA mammary carcinogenesis [54]. In our study, a prominent nuclear and cytoplasmic β-catenin expression in the tumors harvested from DMBA control rats confirms the activation of Wnt/β-catenin pathway at an early stage of chemically induced mammary carcinogenesis in rats. Our results also revealed that PE treatment caused abrogation of Wnt/β-catenin signaling marked by reduced expression of nuclear and cytosolic β-catenin. Since down-modulation of Wnt/β-catenin signaling has been associated with inhibition of cellular proliferation and induction of apoptosis [73], suppression of constitutive activation of Wnt/β-catenin signaling appears to be a possible mechanism of PE-mediated inhibition of cell proliferation and escalation of apoptosis in DMBA-initiated mammary tumorigenesis in rats as we reported earlier [50]. Previously, we have observed that PE exerted similar antiproliferative and proapoptotic effects through modulation of Wnt/β-catenin signaling during chemically induced rat liver carcinogenesis [74]. An ellagitannin-rich pomegranate extract, ellagic acid, and colonic metabolite urolithin have been shown to inhibit canonical Wnt signaling pathway in human 293T cell line [75]. Similarly, a standardized pomegranate extract has been found to modulate several components of Wnt//β-catenin signaling during chemical rat colon carcinogenesis [76].

Cyclin D1, a cofactor for ER action, contributes to mammary tumorigenesis, by the regulation of proliferation and differentiation [77,78]. The mRNA and protein levels of cyclin D1 have been found to be upregulated in more than 50% of the breast cancers and cyclin D1 represents one of the most commonly overexpressed proteins in this cancer [79]. Cyclin D1, a known target for ER, has been reported to be overexpressed favorably in ER-positive breast cancer [79]. In our current study, a substantial expression of intratumor cyclin D1 in DMBA control rats underscores the fundamental role of this cell cycle regulatory protein in DMBA-initiated rat mammary tumorigenesis, supporting previous observations [80,81,82]. A radical reduction of cyclin D1 protein expression due to PE treatment has been observed in our study which indicates that reversal of DMBA-induced dysregulation of a critical cell cycle checkpoint may be one of the possible molecular mechanisms of PE-mediated suppression of mammary tumorigenesis. Our findings also suggest that cyclin D1 may be a potential target of pomegranate bioactive constituents for the chemoprevention of breast cancer. Since cyclin D1 is a β-catenin-regulated gene [83] and PE downregulated cyclin D1 in the same manner as β-catenin, our data confirm interference of Wnt/β-catenin signaling by PE during chemical rat mammary carcinogenesis. Previously, we [74] and other investigators [76] reported pomegranate extract-mediated inhibition of cyclin D1 during experimentally induced rat hepatocarcinogenesis and colon carcinogenesis, respectively.

The identification of specific bioactive phytochemicals of PE responsible for the observed effects in terms with various end-point biomarkers are not evident at this time and requires additional studies. Several pomegranate phytochemicals present in the PE used in this study exhibited synergistic effects in suppressing growth of tumor cells [84,85]. Emerging evidence suggests that plant phytochemicals exert cancer preventive and anticancer effects when they are used in combination rather than individually [86,87]. Accordingly, it is likely that pomegranate phytochemicals may confer the observed activities via promotion of multifactorial effects utilizing chemical synergy.

## 4. Materials and Methods

### 4.1. Materials

PE, a proprietary formulation consists of pomegranate aqueous phase extract and seed oil, was purchased from Rimonest Ltd. (Haifa, Israel). We have previously published a detailed description of the preparation of this product [88]. The chemical analyses of this formulation indicated the presence of mixed octadecatrienoic acids, sterols and steroids, especially 17-α-estradiol, and tocol and γ‑tocopherol in the lipid phase and caffeic acid, corilagin, ellagic acid, ferulic acid, gallic acid, 5-hydroxymethylfurfural, protocatechuic acid, punicalagins (A and B) and *trans*-*p*-coumaric acid in the aqueous phase [88]. The chemical carcinogen DMBA was procured from Sigma-Aldrich (St. Louis, MO, USA). Primary antibodies, such as rabbit polyclonal ER-α (sc-542), ER-β (sc-8974), β-catenin (sc-7199), and cyclin D1 (sc-753) as well as rabbit ABC staining system (sc-2018) were obtained from Santa Cruz Biotechnology (Santa Cruz, CA, USA).

### 4.2. Animals and Experimental Design

Mammary tumor samples for this work were harvested from our previously completed chemopreventive study [50] based on an animal protocol approved by the Institutional Animal Care and Use Committee of Northeast Ohio Medical University (Rootstown, OH, USA). In brief, female Sprague-Dawley rats (Harlan Laboratories, Indianapolis, IN, USA) were divided into six groups. Two groups (groups A and B) were maintained on a basal diet (LabDiet, St. Louis, MO, USA) *ad libitum*, whereas the remaining four groups (groups C, D, E and F) were fed with PE *per os* (p.o.) three times a week (Monday-Wednesday-Friday) in addition to have access to the aforementioned basal diet. Three doses of PE were used for this work: 0.2 g/kg (group C), 1.0 g/kg (group D) and 5.0 g/kg (groups E and F). After the aforementioned treatment period of 2 weeks, mammary tumorigenesis was initiated in all animals belonging to groups B, C, D and E by a single oral administration (p.o.) of DMBA at a dose of 50 mg/kg body weight. Oral treatment of rats with PE in groups C, D, E and F were continued for another 16 consecutive weeks following DMBA administration, *i.e.*, a total period of 18 weeks. After this period, all animals were sacrificed and mammary tumor samples from various DMBA-exposed animals (groups B, C, D and E) were harvested and fixed in 4% paraformaldehyde for immunohistochemical analysis.

### 4.3. Immunohistochemical Analysis

Serial tumor sections, approximately 15-μm thick, were cut using a microtome and stored at −80 °C freezer. For immunohistochemical studies, we used similar regions/locations of tumor mass from each group to prepare tissue sections. We also tried to select tumors with similar size to the extent possible. However, most of the tumors excised from animals treated with medium or high dose of PE had smaller sizes as reported earlier [50]. Intratumor expressions of ER-α, ER-β,β-catenin and cyclin D1 were determined by immunohistochemistry. In short, frozen tissue sections were thawed, air dried for 30 min, and subjected to antigen retrieval by immersing in sodium citrate buffer (10 mM, pH 6.0) at a temperature up to 80 °C for 10 min. Endogenous peroxidases were blocked by 1% H_2_O_2_ (5 min) followed by washing the sections with phosphate-buffered saline (PBS) for 5 min. Tissue sections were then treated with blocking solution for 1 h followed by washing with PBS and incubation overnight (at 4 °C) with primary antibodies (ER-α, ER-β,β-catenin or cyclin D) at a dilution of 1:100. After several washes, tissue sections were treated with horseradish peroxidase-conjugated goat anti-rabbit secondary antibody (1:200) for 30 min at room temperature and then with 3,3’-diaminobenzidine tetrahydrocholoride solution to observe the antigen-antibody complexes. Finally, tumor sections were slightly counterstained with Gill’s hematoxylin solution, air dried, and mounted using DPX (Electron Microscopy Sciences, Hatfield, PA, USA). Various sections of slides were chosen randomly and visualized under a light microscope (BX43, Olympus, Center Valley, PA, USA) which was used to capture representative images. An immunopositive tumor cell expressing an antigen was identified based on brown staining. At least 1000 tumor cells/animal were analyzed. Quantitative results were expressed as percentage of immunopositive cells.

### 4.4. Statistical Analysis

Results are presented as mean ± standard error of the mean (SEM). Significant differences among various treatment groups were determined by one-way ANOVA. *Post hoc* analysis was performed by the Student-Neuman-Keuls test. A *p* value less than 0.05 was considered to be statistically significant. All analyses were performed using commercial software SigmaStat 3.1 (Systat Software, Inc., San Jose, CA, USA).

## 5. Conclusions

Based on results presented here, we conclude that PE abrogates the expression of ER-α and ER-β during DMBA-inflicted mammary tumorigenesis in rats. PE also averts cytosolic stabilization, accumulation, and nuclear translocation of β-catenin, an essential transcriptional cofactor for Wnt/β-catenin signaling. Moreover, PE downregulates the expression of cyclin D1, a downstream target for both ER and Wnt signaling pathways. Based on our earlier study, we have demonstrated a striking mammary tumor-inhibitory effect of PE with concomitant antiproliferative and apoptosis-inducing activities under the same experimental conditions [50]. Hence, the current results together with our previous findings indicate that concurrent disruption of ER and Wnt/β-catenin signaling cascades possibly contributes to antiproliferative and proapoptotic effects involved in PE-mediated prevention of DMBA-initiated mammary carcinogenesis in rats. These encouraging preclinical results coupled with a safety profile may facilitate the development PE as a chemopreventive drug to reduce the risk of breast cancer.
